# Retracted: Influence of DPYD Genetic Polymorphisms on 5-Fluorouracil Toxicities in Patients with Colorectal Cancer: A Meta-Analysis

**DOI:** 10.1155/2020/9470819

**Published:** 2020-11-14

**Authors:** Gastroenterology Research and Practice

Gastroenterology Research and Practice has retracted the article titled “Influence of DPYD Genetic Polymorphisms on 5-Fluorouracil Toxicities in Patients with Colorectal Cancer: A Meta-Analysis” [1]. This article is one of a series of very similar meta-analyses written by different authors that were published in 2014 and 2015, featuring characteristic phrases [2]. Overlaps of wording with these articles are concentrated in the Materials and Methods and Results sections, and a paragraph in the Discussion.

Two similar meta-analyses were not discussed [3, 4]. The authors could not be contacted.
